# Liver transplantation for hepatic trauma: Discussion about a case and its management

**DOI:** 10.4103/0974-2700.76828

**Published:** 2011

**Authors:** Charles Honoré, Arnaud DeRoover, Nathalie Gilson, Olivier Detry

**Affiliations:** Abdominal, Senologic, Endocrine and Transplantation Surgery Department, CHU Liège, Liege, Belgium

**Keywords:** Liver trauma, transplantation, damage control surgery

## Abstract

Liver transplant for trauma is a rare condition with 19 cases described in the literature. We report the case of a 16-year-old patient who suffered a gradeV liver injury with a vena cava tear after a car crash. After a computerized tomography (CT) scan, the patient was directly sent to the operating room where the surgeon performed a right hepatectomy extended to segment IV with a venous repair under discontinued hilar clamping. On day five, the patient developed acute liver failure and was put on an emergency transplant waiting list. He had a successful liver transplant 2 days later. Fifteen months after his transplant, the patient is alive and asymptomatic. This case report focuses on the patient’s initial management, the importance of damage control surgery and the circumstances which finally led to the transplant.

## INTRODUCTION

Blunt liver injuries are common in civilian practice. Non operative management (NOM) is the standard of care with a successful outcome of 83–90%.[1,2] Only partial or non-responders to this treatment are eligible for surgery. In this situation, because trauma patients die from their metabolic failure rather than from incomplete operative repair, damage control surgery was developed 20 years ago. The principle is to stop the bleeding, resuscitate and do the final repair in three individual steps.[3–5] At the end of the line, liver transplant after trauma has already been reported in 19 patients.[7–9] One patient had a liver transplant for trauma in our hospital. We discuss in this article the injury’s initial management, the pitfalls of surgery and the circumstances which eventually led to the transplant.

## CASE REPORT

A 16-year-old patient was admitted to a regional hospital (Level III trauma center) after a car crash. On arrival, the patient was calm, responsive and oriented. His main complaint was diffuse abdominal pain. He had normal hemodynamic parameters. A full-body computerized tomography (CT) scan revealed a gradeV liver injury with massive hemoperitoneum and bilateral pulmonary contusions. On his way back to the Emergency Room (ER), the patient’s systolic blood pressure dropped to 60 mmHg with a sudden increased cardiac pulse. The on-call surgeon was called and he decided to directly send the patient to the operating room (OR). A median laparotomy revealed a complete rupture of the right liver lobe extending to the segment IV with a suspected tear of the right hepatic vein. After nearly 5 min of unsuccessful attempts of direct hemostasis including a Pringle maneuver and direct compression, the surgeon decided to do a right hepatectomy extended to segment IV under discontinued hilar clamping (15/5΄). During the procedure, the surgeon discovered an extension of the right hepatic vein’s tear to the inferior vena cava which was sutured. The venous repair greatly increased the operative time. Total hepatic warm ischemia time was 120 min (eight clampings). Total blood losses were estimated at 20 l. The patient received 22 units of red blood cells (RBC) and 7 units of fresh frozen plasma (FFP) during the operation. Normal hemodynamic parameters were only restored when the opening of the vena cava was closed after about 70 min. At the end of the resection, the patient was in a major coagulopathy with a diffuse intractable bleeding from raw surfaces, which was managed with perihepatic packing. Two abdominal drains were placed before wall closure. The patient was transferred to the intensive care unit (ICU) where his metabolic failure was quickly corrected. The first four postoperative days (POD) were uneventful, but on day five, hepatic, renal and hemostatic biological functions quickly deteriorated. At the same time, abdominal drainage became bloody, even with FFP and platelet transfusion. Hemodynamic status impairment appeared and required continuous vasopressor infusion. The decision was made to transfer the patient in our hospital (Level I trauma and transplant center). On arrival, the patient was directly taken to the OR because of a suspected abdominal compartment syndrome (ACS). After removing the clotted blood and the sponges, the liver looked swollen, discolored, and was hard to the touch. Global visceral edema was so immense that the abdominal wall closure required a silo. After transfer to the ICU, the patient’s renal and liver functions kept deteriorating (INR 1.5, quick 51%, factor V 42%, bilirubin 12.79 mg/ml, albumin 40 mg/dl, creatinine 4.1 mg/dl, pH 7.36) and he became anuric. A grade 4 encephalopathy was confirmed on the EEG. The diagnosis of acute liver insufficiency was established after the Clichy’s criteria[10]. The MELD score was 34. The patient was put on an emergency waiting list for a liver transplant. During the waiting time, the patient was put under Molecular Adsorbents Recirculation System (MARS^®^) and continuous veno-venous hemodialysis (CVVH). During the waiting time, the patient received 25 units of FFP, 11 units of RBC and 2 units of platelets. After 2 days, a cadaveric liver became available. The transplant was performed with a cava replacement. The patient had a caval stenosis at the level of the resected right and median hepatic veins with a thrombus extending to the iliac bifurcation. This prevented our usual use of the “piggy-back” technique for venous anastomosis. The upper anastomosis had to be done inside the pericardium. The visceral edema had decreased, so direct wall closure was possible. Anticoagulation was started on POD one because of the thrombus. The patient was taken back to the OR on POD two because of an intractable bloody drainage, but no active bleeding site was found. We decided to stop the anticoagulation and the drainage became serous. The CVVH was stopped on POD seven after the patient’s renal function fully recovered. The patient was extubated on POD eight and discharged from the ICU on POD 12. A pulmonary embolism was discovered on a control CT scan and was treated with a re-anticoagulation. A symptomatic pleural effusion was surgically drained on POD 30. The patient’s final discharge from the hospital was given on POD 42. His anticoagulation was stopped 8 months after the transplant. The patient is currently alive, back to school and asymptomatic 15 months after his transplant.

## DISCUSSION

In liver trauma care, non operative management (NOM) is now the standard treatment for hemodynamically stable patients, whatever the injury severity scale. It decreases abdominal infection, transfusion rate and hospital stay.[1] In this case, this option was inappropriate because of the patient’s hemodynamic instability. We had no information on the patient’s initial fluid management.

In patients requiring an emergent laparotomy (i.e., non responder to fluid therapy),[11] damage control surgery (DCS) is the established standard care. DCS is based on a damage control trilogy including an abbreviated operation (i.e., stop the bleeding and close), an ICU resuscitation (i.e., correct the metabolic failure) and a return to the OR for the definitive operation once the hemodynamic and metabolic status are back to normal (usually 24–72 h after the trauma). The aim of damage control surgery is to avoid the vicious cycle of hypothermia, acidosis and coagulopathy called the lethal triad (LT).[12] The basic damage control technique for a severe liver injury with massive bleeding is the perihepatic packing.[13] This technique is superior in postoperative outcome to more invasive procedures (clamping, hepatectomy, direct coagulation, ligation, etc.), but the decision must be made quickly before the LT occurs.[3,4] In this case, the surgeon was an expert in hepatobiliary surgery and justified the hepatectomy because the hepatic rupture had already nearly completed the resection and the right liver lobe did not look viable anymore. This case also illustrates the frequent association of ACS in major abdominal trauma.[13] The second part of this case report is focusing on the patient’s hepatocellular insufficiency requiring in the end, a liver transplant. In this case, the association of a large hepactectomy with hilar clamping and a hemorrhagic shock probably induced irreversible ischemic injuries of the liver. Emergency liver transplant has been performed in the past in a two-stage procedure for intractable hemorrhage treated with total hepatectomy in eight patients as a desperate measure. The postoperative mortality rate was 75%.[6] Since the uprising of DCS, liver transplant for liver trauma became anecdotic and 11 cases have been described mainly for postoperative hepatic insufficiency and life-threatening postreperfusion syndrome with a postoperative mortality of 20%.[7–9] The transplant decision is difficult because usual criteria are not validated, liver’s potential recovery is difficult to evaluate and sepsis and head injuries often associated, complicating the decision because of their own prognosis.[7–9]

## CONCLUSION

Emergent liver transplantation after liver trauma is a last resort option only suggested when all other means failed. Appropriate trauma care and especially good application of damage control surgery procedures should avoid its requirement in most situations.[14]
